# Impairment of Respiratory Chain Function and Involvement of Alternative Respiratory Pathway in Mitochondria of Potato Tubers Infected by *Pectobacterium* *carotovorum* subsp. *carotovorum*

**DOI:** 10.3390/foods11111574

**Published:** 2022-05-27

**Authors:** Minzhi Ma, Suleman Muhammad, Jiangong Duan, Lu Bai, Hongyu Li

**Affiliations:** Institute of Microbiology, School of Life Sciences, Lanzhou University, Tianshui Road No. 222, Lanzhou 730000, China; mamzh@lzu.edu.cn (M.M.); suleman2017@lzu.edu.cn (S.M.); jgd@lzu.edu.cn (J.D.); bailu@lzu.edu.cn (L.B.)

**Keywords:** *Solanum tuberosum* L., *Pectobacterium carotovorum* subsp. *carotovorum*, alternative respiratory pathway, mitochondrial respiratory chain function, mitochondrial membrane potential

## Abstract

The significance of alternative respiratory pathway (AOXs) during the interaction between soft rot bacteria (*Pectobacterium carotovorum* subsp. *carotovorum*, (*Pcc*.)) and potato tubers is well-defined. However, the role of the AOXs in impaired mitochondrial respiratory chain function during the *Pcc.* infection is yet to be studied. In this study, the results show that with the aggravation of infection of *Pcc.*, the capacity for alternative respiration in mitochondria of potato tubers increased gradually. The mitochondrial membrane potential increased more significantly after infection with *Pcc.* when the AOXs in potato tubers was partially blocked using salicylhydroxamic acid (SHAM) beforehand. In addition, the activity of complex III decreased more drastically while the activity of complex IV increased more significantly in the partial absence of the AOXs in the mitochondria. Furthermore, the mitochondrial endogenous respiration, mitochondrial respiratory state 3 and respiratory control rate (RCR) decreased more significantly and the value of RCR reached around 1.0 with the aggravation of infection of *Pcc.* in the partially absence of AOXs in the mitochondria.

## 1. Introduction

Potato (*Solanum tuberosum* L.) is the fourth-ranked staple food crop worldwide after wheat, rice, and maize, with a great impact on world agricultural economic stability and food security [1,2]. Potato tubers are not only a rich source of nutrients to provide energy for people, but also have the positive effects of improving anti-inflammatory and anticancer activities for the human body [3,4,5]. However, one of the main causal agents affecting the growth and post-harvest storage of potato tubers is the occurrence of bacterial soft rot disease [6]. Bacterial soft rot disease caused by *Pectobacterium carotovorum* subsp. *carotovorum* (*Pcc*.), is a major disease leading to severe losses of potato tubers during transport and storage [7,8].

During plant-pathogen interactions, an obvious feature of host plants infected by pathogens is an increase in their respiratory rate, and this phenomenon is accompanied by a series of physiological changes in the mitochondrial respiratory chain of host plants at the cellular level [9,10]. It is well known that the mitochondrial respiratory chain is one of the key components of cellular respiration, it is also one of the earliest and most sensitive pathways involved in cell impairment [11]. Involvements of mitochondrial respiration in plant defense responses induced by biotic factors have been implicated in some studies in which the mitochondrial respiratory state 3 or 4 and complex enzymes of the mitochondrial respiratory chain are changed, which can affect plant growth and physiological function [12,13]. More importantly, the change of respiratory rate in the mitochondria of host plants during their interaction with pathogens is mainly caused by the operation of the AOXs in the mitochondrial respiratory chain. 

In contrast to that of animal mitochondria, plant mitochondria possess a unique respiratory pathway, the cyanide-insensitive alternative pathway (AOXs) that is catalyzed by the alternative oxidase, which is located in the mitochondrial inner membrane and acts as a terminal oxidase in mitochondrial electron transport respiration [14,15]. During plant-pathogen interactions, the operation level of the AOXs in host plants are enhanced in response to pathogen attack [16,17,18,19]. It is also believed that the presence of the AOXs allows the flexibility of plant respiratory metabolism, especially under environmental stress [20]. The key role of the AOXs are to prevent over reduction of the respiratory chain and to maintain mitochondrial respiratory chain function when host plants are subjected to biotic condition stresses [21,22,23]. Studies have shown that the capacity of the AOXs in vivo are increased following specific drug inhibition of the activities of complex III or IV during the growth of tobacco suspension cells [24]. Although the compatible interaction between potato tubers and *Pcc*. is accompanied by the acceleration of respiratory rate in tuber slices [14,25], the changes of mitochondrial respiratory processes and complex activity, as well as the role of the AOXs in the impaired mitochondrial respiratory chain remains unknown. Therefore, the study of the role of the AOXs on mitochondrial respiratory chain function during the interaction between potato tubers and *Pcc.* is critical to understanding more about the AOXs as valuable regulators of mitochondrial respiratory chain function during pathophysiological processes in potato tubers [15].

The objectives of this study were to provide a theoretical insight into the mitochondrial respiratory chain during the interaction between potato tubers and soft rot bacterial infection. We observed the extent of infection of two strains of *Pcc*. in potato tubers by estimating the mitochondrial membrane potential in them and determining the total as well as the alternative respiratory rate and analyzing the activity of the complex III and complex IV during the mitochondrial respiratory chain processes modulated by *Pcc*.S and *Pcc*.L, respectively.

## 2. Materials and Methods

### 2.1. Potato Tubers and Pathogenic Bacteria

The susceptible variety of potato tubers (*Solanum tuberosum* L. cv. Atlantic) was provided by the School of Life Sciences, Gansu Agricultural University. Tubers without obvious defects or physical injuries and of appropriate size and appearance were selected. To maintain the physiological activity, potato tubers were placed in mesh bags, brought to the laboratory within 4 h, and stored in darkness at 20 ± 3 °C, RH 85–90% conditions before further use.

*Pectobacterium carotovorum* subsp. *carotovorum* (*Pcc*.) is a major pathogen of soft rot disease of potato tuber. Two strains of *Pcc*. were selected and named *Pcc*.S and *Pcc*.L, respectively, and were provided by the Microbiology Laboratory of the School of Life Sciences of Lanzhou University, China. These two strains of *Pcc*. were selected for their ability to produce soft rot symptoms on potato tubers, either on detached potato tuber or in inoculated plants. The *Pcc*.S had strong pathogenicity, while the *Pcc*.L had weak pathogenicity to potato tubers [14]. Both *Pcc*.S and *Pcc*.L were cultured in 100 mL liquid Luria–Bertani medium at a shaker with a temperature of 26 ± 1 °C and a speed of 160 rpm/min. Finally, the suspension of the two strains of *Pcc*. were diluted and adjusted to a concentration of 1.0 × 10^8^ CFU/mL with sterile distilled water. 

### 2.2. Experiment Design and Treatments

Salicylhydroxamic acid (SHAM), as a specific inhibitor of the AOXs that can inhibit alternative oxidase activity [26,27], was used in this study. The experiment design was that some of the potato tuber slices were vacuum-infiltrated by 5 mM SHAM (+sham) within 30 min, and another set of the potato tuber slices were without SHAM, or no-SHAM treatment (−sham). 

Healthy potato tubers of the same size were selected. One tuber was soaked in 300 mL 1% sodium hypochlorite solution in a sterile beaker to surface sterilize it for 5 min. This was followed by soaking it in 300 mL 75% ethanol for 1 min in another sterile beaker. The tubers were then rinsed 3–4 times with sterile distilled water, followed by air-drying on a sterile bench. Then, round slices of 6 mm diameter and 1 mm in thickness were cut with a sterile puncher and scalpel from the tubers. 

The potato tuber slices (10 g, diameter 6 mm, thickness 1 mm approximately) were each pretreated with 50 µL 5 mM SHAM in a sterile Petri dish (diam = 90 mm), followed by absorption using vacuum filtration for 30 min to completely absorb the SHAM into the potato tuber slices. After vacuuming, the slices were inoculated with stirring in each Petri dish, respectively, with 10 mL of the two strains of bacterial suspensions for 2, 4, 6, 8, 10, 12 h in a microbial incubator at 26 ± 1 °C, 16 h illumination and 8 h darkness conditions. The control groups (natural aging tuber slices) were placed in a Petri dish with the same amount of sterile distilled water. All experiments were carried out in triplicate. 

### 2.3. Observation and Measurement of Soft Rot Degree of Potato Tuber Slices

The samples of potato tuber slices with each treatment were observed at 2, 4, 6, 8, 10, 12 h after infection with *Pcc*.S and *Pcc*.L, respectively. The extent of the symptoms was evaluated by comparison with the phenotype characteristics of the symptoms caused by *Pcc*.S and *Pcc*.L in potato tuber slices, and by determining the amount of softened tissue in the potato tuber slices. 

The membrane permeability of the samples of potato tuber slices was measured using a conductivity meter (DDS-510) according to the method of Hua et al. [14]. The bacterial suspension was aspirated from the 10 mL test tube with 1.0 g samples of tuber slices, and 3 mL of deionized water was added to the test tube, this conductivity was measured and recorded as P_1_. The conductivity of deionized water without samples of tuber slices was determined and recorded as P_0_. The test tube of each sample was placed in boiling water for 30 min and then removed and kept at 25 °C. The conductivity of each tube was measured again and recorded as P_1_*. The conductivity of deionized water without samples of tuber slices was measured under the same conditions and recorded as P_0_*.

Membrane permeability (*P*%) = [1 − (P_1_ − P_0_)/(P_1_* − P_0_*)] × 100%

### 2.4. Extraction and Separation of Mitochondria

Mitochondria of treated potato tuber slices were extracted according to a method previously described by Hua et al. [14] with modifications. First, 10 g of inoculated potato tuber slices and extraction solution (350 mM mannitol, 250 mM sucrose, 1 mM EDTA, 0.1% Bovine serum albumin (BSA), 10 mM Tris-HCl buffer) were mixed in a 1:2 ratio (*w*/*v*), then the pH was adjusted to 7.2. The homogenate was filtered through a 200-mesh sieve and centrifuged at 1000× *g* for 15 min, and the resulting supernatant was centrifuged at 10,000× *g* for 15 min to yield the mitochondrial pellet. The pellet was washed with mitochondrial washing solution (350 mM mannitol, 250 mM sucrose, 0.1% BSA, 10 mM Tris-HCl buffer, pH 7.2), and the suspension was centrifuged at 250× *g* for 10 min. The supernatant was centrifuged at 9000× *g* for 15 min again. Finally, the mitochondrial pellet was suspended with a washing solution. All the steps were strictly operated on ice to ensure the isolation of high-quality mitochondria preparation. The mitochondrial protein content was determined using the BCA Protein Assay Kit (Beijing Solarbio Science & Technology, Co., Ltd., Beijing, China). 

### 2.5. Measurement of Mitochondrial Membrane Potential

The mitochondrial membrane potential was determined by flow cytometry according to the method of Doherty and Perl [28]. A 500 µL mitochondrial pellet suspension of each treatment was added to a flow tube for staining. Then, 10 µM Rhodamine123 was added into the mitochondrial suspension and incubated at 37 °C for 10 min. The mitochondrial membrane potential was determined by the release of Rhodamine123 from the mitochondria by using flow cytometry. The data were analyzed with FlowJo version 7.5.5 software (Tree Star Inc., Ashland, OR, USA).

### 2.6. Measurement of AOXs in Mitochondria

Aliquots of mitochondria (0.2 mg/mL) were used in all of the respiration measurements using a Clark-type oxygen electrode (Hand-Tech) according to Jacoby et al. [26] with modification. A 50 µL mitochondrial pellet suspension was added to a mixture containing respiratory substrate 10 µL 0.4 M α-ketoglutaric acid and the assay was performed in 1800 µL of the reaction mixture of 300 mM sucrose, 10 mM NaCl, 5 mM KH_2_PO_4_, 2 mM MgSO_4_, 0.1% BSA, and 10 mM TES, pH 7.2. The AOXs rate was defined as the sensitivity of O_2_ uptake to 50 µL 5 mM SHAM. The COX respiratory pathway rate was defined as the sensitivity of O_2_ uptake to 50 µL 1 mM KCN in the presence of 5 mM SHAM. 

### 2.7. Measurement of Complex III and Complex IV Activity of Mitochondrial Respiratory Chain

The complex III activity was measured by monitoring the reduction of cytochrome c (20 µM) at 550 nm according to a method of Jacoby et al. [26] with modification. A volume of 180 µL reaction mixture consisted of 25 mM potassium phosphate (pH 7.2), 0.5 mM EDTA, 5 mM MgCl_2_, 2 mM KCN, 2 µg/mL rotenone, 20 mM succinate, and 0.1% BSA. Then, 0.4 µL 25 µM cytochrome c and 1 µL 100 µM ubiquinol were added to the assay medium, and the nonenzymatic rate was recorded for 1 min. Then, the 20 µL mitochondrial freeze-thawing liquid was added and the increase in absorbance was measured and recorded for 3 min. 

The complex IV activity (cytochrome c oxidase activity) was measured by oxidation of cytochrome c (20 µM) at 550 nm in a reaction medium containing 180 µL 25 mM potassium phosphate (pH 7.2) and 0.4 µL 25 µM reducing cytochrome c. Then, 20 µL mitochondrial freeze-thawing liquid was added into the reaction medium and the reduction of absorbance was recorded for 3 min according to the method of Jacoby et al. [26] with modification. 

### 2.8. Measurement of Mitochondrial Respiratory Chain Function

Aliquots of mitochondria (0.2 mg/mL) were used in all of the respiration measurements using a Clark-type oxygen electrode (Hand-Tech) according to Jacoby et al. [26] with modification. A 50 µL mitochondrial suspension was added to a mixture containing a respiratory substrate of 10 µL 0.4 M α-ketoglutaric acid. The assay was performed in a reaction mixture with 0.35 M mannitol, 0.25 M sucrose, 10 mM KCl, 0.2 mM EDTA, 5 mM MgCl_2_, 10 mM Tris-HCl buffer (pH 7.2), 5 mM phosphate buffer (pH 7.2). Then, 0.5 mL mitochondrial suspension and 50 µL respiratory substrate were added into the reaction chamber, the oxygen absorption rate reached equilibrium, and then 1.2 µM ADP was added, the oxygen absorption rate was called state 3. When the phosphorylation reaction substrate ADP was consumed, the oxygen absorption rate of mitochondrial decreased automatically, which was in state 4. The respiratory control rate (RCR) was calculated using the ratio of state 3 and state 4 respiratory rates. 

### 2.9. Statistical Analysis

The effects of infection status, pathogenicity, membrane permeability, mitochondrial total respiration, capacity of alternative respiration, Valt’/Vtotal, mitochondrial state respiration, respiratory control ratio, complex III and complex IV activity, and mitochondrial membrane potential were analyzed by three-way ANOVA (linear mixed-effect models). Data for two strains of *Pcc.* inoculations with the same treatment times were analyzed, and the mean differences determined using the independent-sample *t*-test with SPSS version 22.0 software for (SPSS Inc., Chicago, IL, USA).

## 3. Results

### 3.1. Infection Degree of Pcc.L and Pcc.S on Potato Tuber Slices

**Figure 1:**
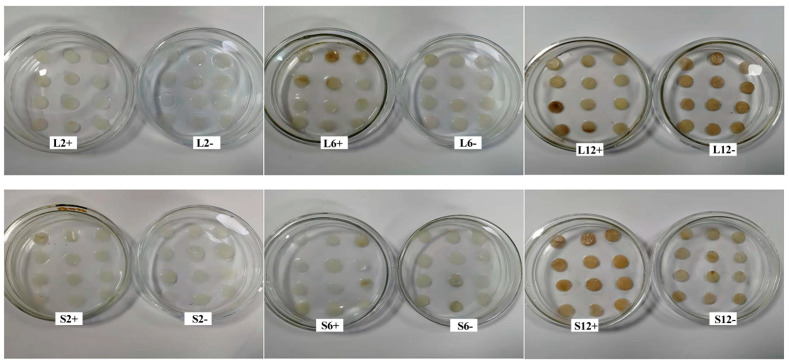
The effect of *Pcc*.L and *Pcc*.S treatments on the infection degree of potato tuber slices. “L” and “S” indicate pathogenic bacteria of *Pcc*.L and *Pcc*.S, respectively. “2, 6, 12” indicate post-inoculation time (h). “+” and “−” indicate salicylhydroxamic acid pretreatment and without salicylhydroxamic acid treatment, respectively.

The results of the degree of infection of soft rot bacteria on potato tuber slices showed that there was stronger pathogenicity and a higher degree of rot in potato tuber slices infected by *Pcc*.S than in those infected by *Pcc*.L, whether in the SHAM pretreated groups (+sham) or in the no-SHAM treatment groups (−sham) (Figure 1). The membrane permeability of tuber slices infected by *Pcc*.S and *Pcc*.L in the SHAM pretreated groups were 80.98% and 70.41%, respectively, and were 65.80% and 60.20% at 12 h after infection, respectively (Table 1). In addition, the membrane permeability of tuber slices in the SHAM pretreated groups were 1.15 times and 1.09 times as great as that of in the no-SHAM treated groups infected by *Pcc*.L and *Pcc*.S, respectively.

### 3.2. Mitochondrial Membrane Potential 

**Figure 2:**
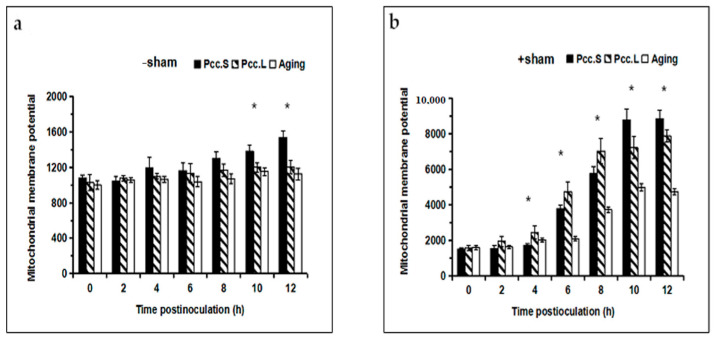
The effect of *Pcc*.L and *Pcc*.S treatments on mitochondrial membrane potential of tuber slices in the no salicylhydroxamic acid treated group (**a**) and that of the salicylhydroxamic acid pretreated group (**b**). Aging indicates naturally aged potato tuber slices as control. Bars with * (*t*-test) were significantly different at *p* < 0.05 between *Pcc*.S and *Pcc*.L. The standard errors (Vertical bars) of three replicates are shown.

The value of the mitochondrial membrane potential showed a continuous increasing trend when infected by *Pcc*.L and *Pcc*.S, both in the SHAM pretreated groups and in the no-SHAM treated groups, but the value of the mitochondrial membrane potential in the SHAM pretreated groups was more than that of the no-SHAM treated groups. There were significant differences (*p* < 0.05) between the mitochondrial membrane potential of tuber slices after infection with *Pcc*.S and *Pcc*.L from 4 h to 12 h, respectively (Figure 2b). But there were significant differences (*p* < 0.05) between the mitochondrial membrane potential of tuber slices after infection with *Pcc*.L and *Pcc*.S at 10 h and 12 h. (Figure 2a). In addition, the value of the mitochondrial membrane potential in the SHAM pretreated groups was 6.32 times and 5.86 times as great as that in the no-SHAM treated groups at 12 h after infection with *Pcc*.L and *Pcc*.S, respectively. 

### 3.3. Mitochondrial Alternative Respiratory Pathway

**Figure 3:**
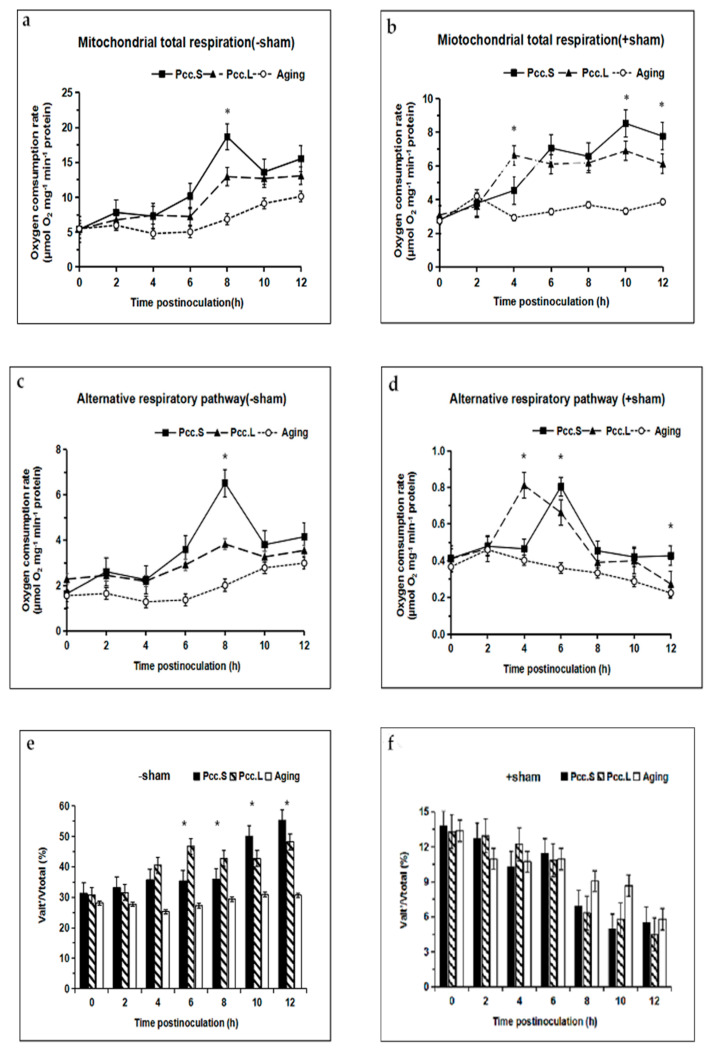
Effect of *Pcc*.L and *Pcc*.S treatments on mitochondrial total respiratory rate, alternative respiratory rate and Valt’/Vtotal of tuber slices in no salicylhydroxamic acid treated group (**a**,**c**,**e**) and those of the salicylhydroxamic acid pretreated group (**b**,**d**,**f**). Aging indicates naturally aged potato tuber slices as control. Bars with * (*t*-test) were significantly different at *p* < 0.05 between *Pcc*.S and *Pcc*.L. The standard errors (Vertical bars) of three replicates are shown.

Compared with the control, the total respiratory rate of mitochondria increased in the SHAM pretreated groups and in the no-SHAM groups after infection with two strains of *Pcc*. The total respiratory rate of mitochondria infected with *Pcc*.S at 8 h was significantly higher (*p* < 0.05) than that infected by *Pcc*.L (Figure 3a), and there were significant differences (*p* < 0.05) between total respiratory rate after infection with *Pcc*.S and *Pcc*.L at 4 h, 10 h, and 12 h.

In the no-SHAM treated groups, the actual respiratory rate of the AOXs increased in both mitochondria of tuber slices infected by *Pcc*.L and *Pcc*.S, and the actual alternative respiratory rate of mitochondria infected by *Pcc*.S at 8 h was significantly higher (*p* < 0.05) than that of mitochondria infected by *Pcc*.L (Figure 3c). In the SHAM pretreated groups, the actual alternative respiratory rate of mitochondria had increased and then decreased in both mitochondria of tuber slices infected by *Pcc*.L and *Pcc*.S. There were significant differences (*p* < 0.05) between actual alternative respiratory rates infection with *Pcc*.S and *Pcc*.L at 4 h, 6 h, and 12 h after infection (Figure 3d).

Compared with the control group, the ratio of alternative respiratory rate to total respiratory rate (Valt’/Vtotal) increased after infection with two strains of *Pcc*. in the no-SHAM treated groups, and there were significant differences (*p* < 0.05) between *Pcc*.S and *Pcc*.L infection from 6 h to 12 h (Figure 3e). In the SHAM pretreated groups, the value of Valt’/Vtotal showed a decreasing trend (Figure 3f). The value of Valt’/Vtotal in the no-SHAM treated groups was 10.78 times and 10.03 times as great as that in the SHAM pretreated groups at 12 h after infection with *Pcc*.L and *Pcc*.S, respectively.

### 3.4. Mitochondrial Complex III and Complex IV Activity

**Figure 4:**
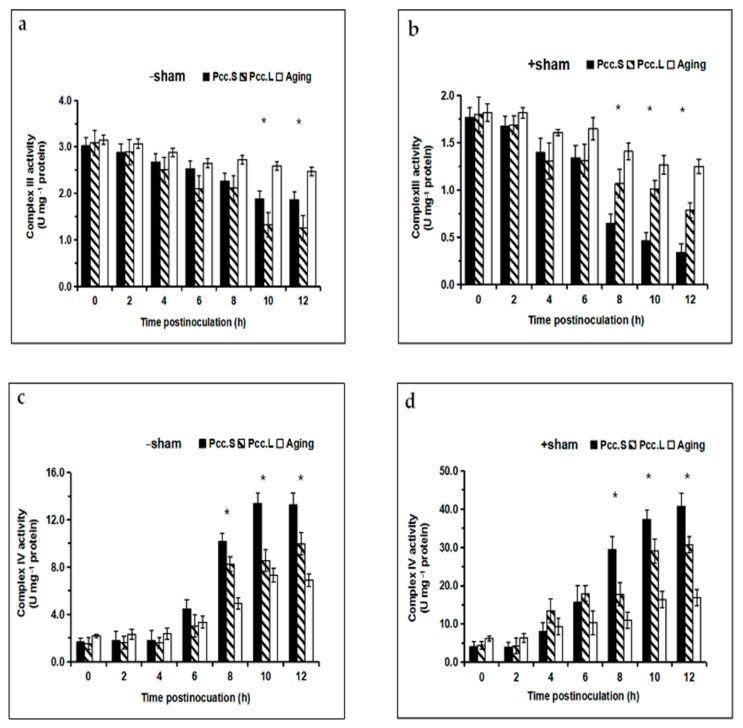
Effect of *Pcc*.L and *Pcc*.S treatment on mitochondrial complex III and complex IV activity of tuber slices in the no salicylhydroxamic acid treated group (**a**,**c**) and those of the salicylhydroxamic acid pretreated group (**b**,**d**). Aging indicates naturally aged potato tuber slices as control. Bars with * (*t*-test) were significantly different at *p* < 0.05 between *Pcc*.S and *Pcc*.L. The standard errors (Vertical bars) of three replicates are shown.

We observed a substantial decline in the activity of complex III in the mitochondria of infected tuber slices, both in the SHAM pretreated groups and in the no-SHAM treated groups. Compared with the control, the activity of complex III exhibited the earliest and strongest action in response to *Pcc*.L infection in the SHAM pretreated groups, and there were significant differences (*p* < 0.05) between *Pcc*.S and *Pcc*.L infection from 8 h to 12 h (Figure 4b). However, the activity of complex III showed the strongest response to *Pcc*.S infection in the no-SHAM treated groups, and there were significant differences (*p* < 0.05) between *Pcc*.L and *Pcc*.S infection from 10 h to 12 h (Figure 4a). The activity of complex III in the SHAM pretreated groups was 35.94% and 83.33% lower than that of in the no-SHAM treated groups at 12 h after infection with *Pcc*.L and *Pcc*.S, respectively.

The activity of complex IV increased in mitochondria throughout the infection phase, but the enhancement of complex IV activity in the SHAM pretreated groups was more than that of in the no-SHAM treated groups (Figure 4c,d). The activity of complex IV showed the strongest susceptibility in response to *Pcc*.S infection, and there were significant differences (*p* < 0.05) between *Pcc*.S and *Pcc*.L infection from 8 h to 12 h, both in the SHAM pretreated groups and in the no-SHAM treated groups (Figure 4c,d). The activity of complex IV in the SHAM pretreated groups was 66.17% and 78.71% higher than that of in the no-SHAM treated groups at 12 h after infection with *Pcc*.L and *Pcc*.S, respectively.

### 3.5. Mitochondrial Respiratory Chain Function

**Figure 5:**
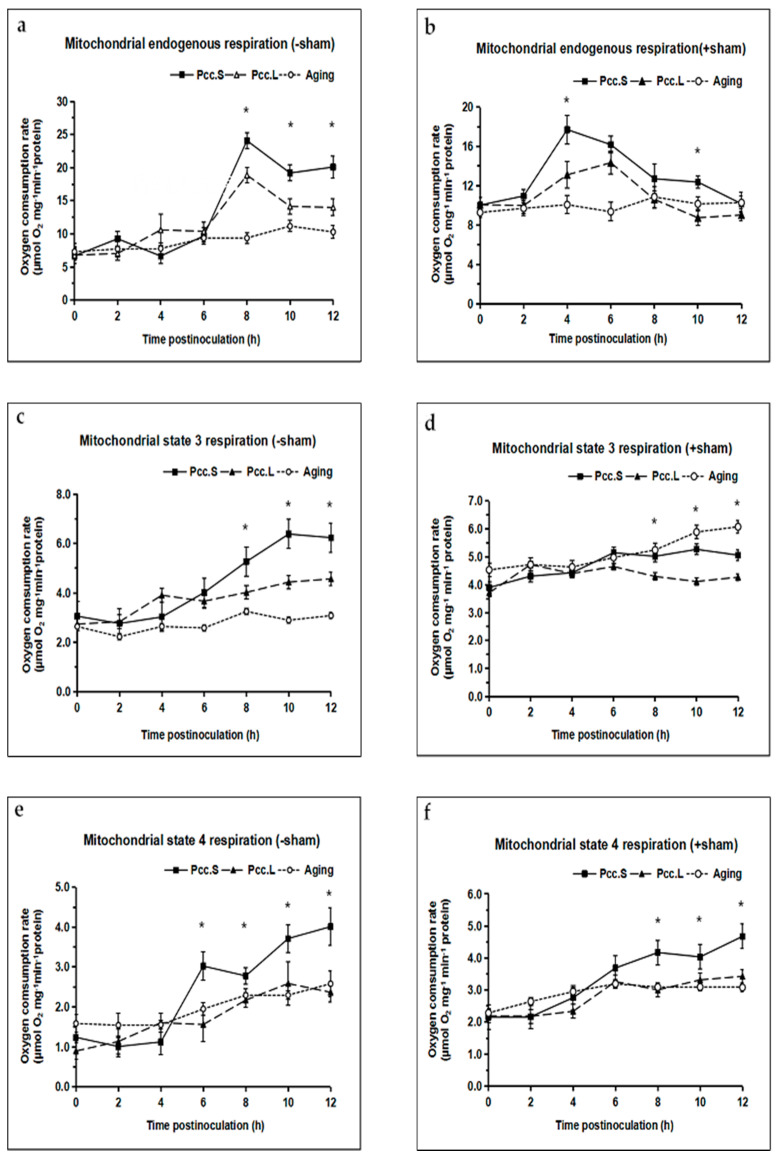

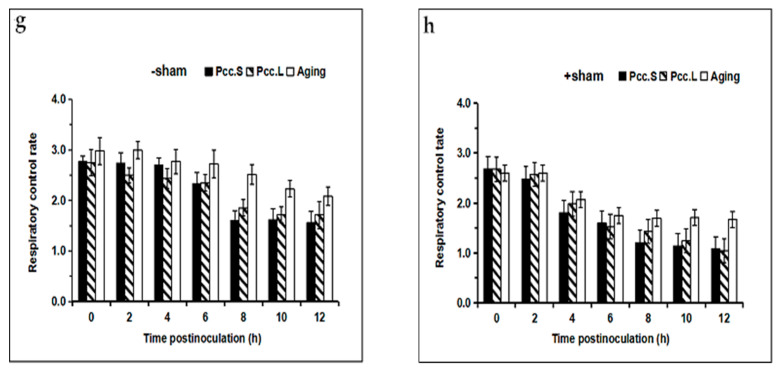
Effect of *Pcc*.L and *Pcc*.S treatment on mitochondrial endogenous respiration, mitochondrial state 3, state 4 and the relative control rate of tuber slices in the no salicylhydroxamic acid treated group (**a**,**c**,**e**,**g**) and those of the salicylhydroxamic acid pretreated group (**b**,**d**,**f**,**h**). Aging indicates naturally aged potato tuber slices as control. Bars with * (*t*-test) were significantly different at *p* < 0.05 between *Pcc*.S and *Pcc*.L. The standard errors (Vertical bars) of three replicates are shown.

The endogenous respiratory rate of mitochondria showed an increasing trend infected by *Pcc*.S and *Pcc*.L as compared to the control group. There were significant differences (*p* < 0.05) between the mitochondrial endogenous respiratory rate at 8 h, 10 h and 12 h after inoculation *Pcc*.S and *Pcc*.L (Figure 5a). However, in the SHAM pretreated groups, the mitochondrial endogenous respiratory rate increased firstly and then declined induced by *Pcc*.S and *Pcc*.L, respectively (Figure 5b). 

In the no-SHAM treated groups, the respiratory state 3 and state 4 of mitochondria began to rise rapidly in the tuber slices infected by two strains of *Pcc*., and there were significant differences (*p* < 0.05) in them between *Pcc*.S and *Pcc*.L infection from 6 h to 12 h, respectively (Figure 5c,e). Unlike the no-SHAM treated groups, the respiratory state 3 of mitochondria changed slowly in the SHAM pretreated groups (Figure 5d). 

The value of RCR displayed a continuous decline trend in mitochondria infected by *Pcc*.L and *Pcc*.S as compared to the control (Figure 5g). However, the value of RCR in the SHAM pretreated groups decreased more than that of the no-SHAM treated groups (Figure 5h). The values of RCR in the SHAM pretreated groups was 38.59% and 30.32% lower than that of in the no-SHAM treated groups at 12 h after infection with *Pcc*.L and *Pcc*.S, respectively.

## 4. Discussion

Potato tubers are easily invaded by soft rot bacteria *Pcc*. in the post-harvest and storage stage. Mitochondria play an important role in potato tuber in response to soft rot disease. The change in the mitochondrial respiratory pathway leads to efficient disease response and may protect the plant from pathogen invasion [15]. This study was conducted to observe the mitochondrial responses during the interaction between potato tubers and soft rot bacteria *Pcc*., and especially the role of the AOXs in the mitochondrial respiratory chain. The infection of potato tuber slices by *Pcc*.L and *Pcc*.S can lead to soft rot in tuber slices and an increase of their membrane permeability at 12 h after infection. It was also found that the symptomatic infection and membrane permeability were higher in the potato tuber slices when the AOXs were inhibited using SHAM, suggesting that the presence of the AOXs alters the disease susceptibility of potato tubers. This result is similar to that of a study by Li et al. where they present that soft rot bacterial infection of potatoes in activation of the increase of cyanide-resistant respiration in tubers, and *Pcc*.S can induce more cyanide-resistant respiration in tubers [29]. 

In addition to phenotypic changes, an increase in the respiratory rate of a host plant is a widespread phenomenon during plant-pathogen interaction [10,30]. We determined that the rates of the total respiration and the alternative respiration both increased in response to pathogen infection in the without SHAM treatment group. The capacity of alternative respiration and the value of Valt’/Vtotal were very low and tended to decrease when the AOXs were inhibited by SHAM. This result of an increase of mitochondrial AOXs and of total respiration of tuber slices induced by *Pcc*. is consistent with the results proposed by Simons et al. They showed that the infection of *Arabidopsis* leaves with the virulent tomato strain *Pseudomonas syringae* pv. resulted in an increase in both total respiration and cyanide-resistant O_2_ uptake level [30]. 

The mitochondrial respiratory chain consists of a few other enzyme complexes besides the AOXs. Among them, complex III and complex IV are the key enzymes associated with mitochondrial respiratory chain reactions. Complex III is cytochrome b-c1, which is responsible for electron transfer, while complex IV is the only oxidase in the mitochondrial respiratory chain, and cause the oxidation-reduction reactions of O_2_ leading to the synthesis of two H_2_O molecules ultimately [31,32]. Evidence showed that in the interaction between host plants and pathogens, complex III and complex IV were impaired by pathogens, resulting in mitochondrial respiratory chain dysfunction [33,34]. In this study, the activity of complex III in mitochondria of the tubers declined with the aggravation of *Pcc*. infection regardless of the presence of the AOXs, suggesting that the mitochondrial respiratory chain was disrupted by *Pcc*. which led to the mitochondrial respiratory chain dysfunction. It was also found that the activity of complex III declined, while the activity of complex IV increased when the AOXs were partially blocked with SHAM. This result suggests that the AOXs were able to correct pathological states associated with respiratory impairment, notably those affecting complex III and complex IV, and to maintain the function of the mitochondrial respiratory chain as reported by Szibor et al. and Giordano et al. in their respective studies [35,36]. 

We also found that in the presence of the AOXs, the mitochondrial endogenous respiration showed a trend of increasing rapidly with infection of pathogenic bacteria. This result showed that the increased mitochondrial endogenous respiration of the host plant was mainly caused by the operation of the AOXs [10]. Generally, states 3 and 4 are studied to evaluate mitochondrial function. State 3 reflects the substrate permeability of the mitochondrial inner membrane and the operation of the respiratory chain. State 4 reflects the permeability of the mitochondrial inner membrane, which is regulated by the mitochondrial membrane potential [26]. The RCR reflects mitochondrial membrane integrity and mitochondrial function state, as well as the degree of oxidative phosphorylation coupling [37]. In this study, we found that the value of RCR decreased with the increase of the infection degree of soft rot bacteria, but the value was greater than 1.5 in the presence of the AOXs. The value of RCR decreased rapidly and was close to 1.0 when the AOXs was partially blocked. This result suggests that the uncoupling degree of mitochondrial oxidative phosphorylation was enhanced when the AOXs were inhibited. It further illustrates that the mitochondria were disrupted in varying degree by two strains of pathogenic bacteria, which increased mitochondrial membrane permeability, resulted in the decline of mitochondrial integrity and in a series of membrane dysfunctions, as well as decreased respiratory rate, and finally led to mitochondrial respiratory dysfunction. The results suggest that the AOXs involve and regulate the mitochondrial respiration chain function to maintain host plant metabolism under the pathogenic bacteria stress [20].

## 5. Conclusions

The mitochondrial respiratory chain of potato tubers was impaired by two strains of *Pcc*. to varying degrees, which led to the increase of the AOXs. However, when the AOXs were partially blocked, the mitochondrial membrane integrity and complex III activity was significantly reduced with the increased soft rot symptoms. We conclude that increased AOXs activity can maintain mitochondrial respiratory chain and protect mitochondrial function to a certain extent, so as to alleviate the damage of pathogenic bacteria to potato tubers. This study provides insights for plant breeders to develop varieties with increased AOXs activity to prevent plants from oxidative damage caused by biotic stresses.

## Figures and Tables

**Table 1:** The change of potato tuber slices after inoculation with *Pcc*.L and *Pcc*.S on 12 h.

Treatment	Strains	Pathogenicity	Rot Degree	Color	*p* %
−sham	Aging	−	+	White	49.90 d
	*Pcc*.L	weak	+++	Brown	60.20 c
	*Pcc*.S	strong	++++	Dark brown	65.80 c
+sham	Aging	−	+	White	53.78 d
	*Pcc*.L	weak	++++	Brown	70.41 b
	*Pcc*.S	strong	+++++	Dark brown	80.98 a

+ sham indicates potato tuber slices were pretreated by salicylhydroxamic acid; − sham indicates potato tuber slices without salicylhydroxamic acid. Aging indicates naturally aged potato tuber slices as control. “+” indicates rot degree of potato tuber slice infected by *Pcc*.L and *Pcc*.S. *P* % indicates membrane permeability of potato tuber slices. “a,b,c,d” were significantly different at *p* < 0.05 for the same treatment.

## Data Availability

No new data were created or analyzed in this study. Data sharing is not applicable to this article.
